# Genetic Resistance Determinants in Clinical *Acinetobacter pittii* Genomes

**DOI:** 10.3390/antibiotics11050676

**Published:** 2022-05-17

**Authors:** Itziar Chapartegui-González, María Lázaro-Díez, José Ramos-Vivas

**Affiliations:** 1 Department of Microbiology and Immunology, University of Texas Medical Branch (UTMB), Galveston, TX 77550, USA; 2 Instituto de Agrobiotecnología, CSIC-Gobierno de Navarra, 31192 Mutilva, Spain; marialazarodiez@gmail.com; 3 Department of Project Manager, Universidad Internacional Iberoamericana, Campeche 24560, Mexico; jose.ramos@uneatlantico.es; 4 Research Group on Foods, Nutritional Biochemistry and Health, Universidad Europea del Atlántico, 39001 Santander, Spain; 5 CIBER of Infectious Diseases—CIBERINFEC, Instituto de Salud Carlos III, 28029 Madrid, Spain

**Keywords:** *Acinetobacter pittii*, WGS, MDR, plasmid, nosocomial

## Abstract

Antimicrobial-resistant pathogenic bacteria are an increasing problem in public health, especially in the healthcare environment, where nosocomial infection microorganisms find their niche. Among these bacteria, the genus *Acinetobacter* which belongs to the ESKAPE pathogenic group harbors different multi-drug resistant (MDR) species that cause human nosocomial infections. Although *A. baumannii* has always attracted more interest, the close-related species *A. pittii* is the object of more study due to the increase in its isolation and MDR strains. In this work, we present the genomic analysis of five clinically isolated *A. pittii* strains from a Spanish hospital, with special attention to their genetic resistance determinants and plasmid structures. All the strains harbored different genes related to β-lactam resistance, as well as different MDR efflux pumps. We also found and described, for the first time in this species, point mutations that seem linked with colistin resistance, which highlights the relevance of this comparative analysis among the pathogenic species isolates.

## 1. Introduction

Antimicrobial resistance is a rising problem in public health around the world, being the current cause of millions of deaths annually [1]. Among all these fatalities, over 80% are caused by one of the ESKAPE pathogens (*Enterococcus faecium*, *Staphylococcus aureus*, *Klebsiella pneumoniae*, *Acinetobacter baumannii*, *Pseudomonas aeruginosa*, and *Enterobacter* spp.), in which *A. baumannii* is included. Although *A. baumannii* is considered the most problematic and pathogenic bacteria in its complex (*A. calcoaceticus-baumannii* complex), the species *A. pittii* is increasing in relevance because of the growing number of clinical isolates, which seems to be due to the improvement in molecular identification [2,3,4]. It has been extensively considered that this specie has been under-reported because of the phenotypic similarities with *A. baumannii*, mainly due to the lack of molecular identification in the clinical settings and hence causing its misidentification [5,6,7]. In fact, *A. pittii* isolates harboring carbapenem β-lactamase as NDM-1 have already become a medical concern [4,8,9]. *A. pittii* is a Gram-negative rod responsible for nosocomial infections, with catheter-associated blood infection being the most common manifestation of this pathogen [2,10]. In fact, *A. pittii* infections are more commonly associated with higher comorbidities than other species from that genus, presented for example in patients with malignant neoplasia [11]. As well as *A. baumannii*, these bacteria are difficult to eradicate from hospital settings due to their ability to survive under stressful conditions (e.g., starvation, desiccation), acting also as a reservoir for antimicrobial resistance genes in clinical facilities that could be easily disseminated to other pathogens [10,12,13,14].

This bacteria incidence, besides being considered underestimated, is also variable among countries, being more common in North Europe countries such as Denmark, Ireland, and Germany [11,12]. A prevalence study in Norway showed that this species was the most prevalent one within the complex isolates, comprising almost half of the positive cultures [15].

The main features of *A. pittii* genomic species are the same as those in the closely related species *A. baumannii*, which are sized from 3.7 to 4 Mb, with between 3400 and 3800 encoding genes (depending on strains), a GC content of up to 40% (usually 35–40%), and high ability to acquire new resistance mechanisms through mobile elements [2]. In those genomes, as well as in many pathogenic bacteria, there are a large number of mobile elements, which can carry resistance determinants, genomic islands, and transposable elements. Both the new acquisition and dissemination of antimicrobial resistance genes are mainly due to the role of mobile genetic elements, which can move some sequences in the same bacteria (intracellular DNA) or to a new cell through horizontal gene transfer (intercellular DNA).

Both genetic and virulence determinant information of this species are scarce, and usually extrapolated from the genomic analysis of *A. baumannii*, due to their high genomic similarity [16]. While *A. pittii* is commonly more sensitive to antimicrobial agents than *A. baumannii*, both MDR and XDR strains have been found as a source of ICU outbreaks and nosocomial infections [7,10,12,17]. Therefore, new studies contributing to the understanding of these mechanisms are required. In this work, we present a pan-genome comparison and a detailed analysis of resistance determinants in five clinically isolated *A. pittii* strains.

## 2. Results

### 2.1. Antimicrobial Susceptibility Assays

The MIC values for the clinically tested strains are shown in Table 1, for which *Acinetobacter* breakpoints established by EUCAST were used when available. According to the table, all the tested strains showed resistance against colistin, with the strains HUMV0315 and HUMV6207 showing the lowest value. These data are in accordance with previous reports that showed this species being more usually resistant to colistin than *A. baumannii* [10,11,18,19]. All the strains were also resistant to ampicillin, but only HUMV0315 was resistant to meropenem. All the strains were susceptible to ciprofloxacin and tetracycline, while resistant to erythromycin. The fluoroquinolone gentamicin was effective against five out of the six tested strains, with only the strain HUMV4336 showing resistance to this compound. According to the results, the strain HUMV0315 was the most resistant among the isolates, being resistant against five out of the seven tested antimicrobials, while the HUMV6207 was resistant against four. The rest of the strains were resistant to colistin, ampicillin, and erythromycin. However, because only one compound of each antibiotic class was tested, these *A. pittii* isolates can only be considered MDR (multidrug resistant) [20].

### 2.2. Genomic Assembly and Pan-Genome Analysis

Four strains were submitted to PacBio sequencing (HUMV4336, HUMV6207, HUMV5918, and HUMV6483) for which the final assembly resulted in genomes of 3.95 Mb (HUMV6207), 3.91 Mb (HUMV4336, HUMV5918), and 4.09 Mb (HUMV6483). Due to the high similarity between HUMV4336 and HUMV5918, only the former was also submitted to Illumina sequencing with the other *A. pittii* isolates. In Table 2, the main characteristics of each strain are shown, including the CDS (protein-coding sequences) predicted by the RAST and Prokka software. None of the sequenced genomes had any sequence flagged as poor quality according to the FastQC analysis report, which suggests trustable data was obtained from them.

**Figure 1:**
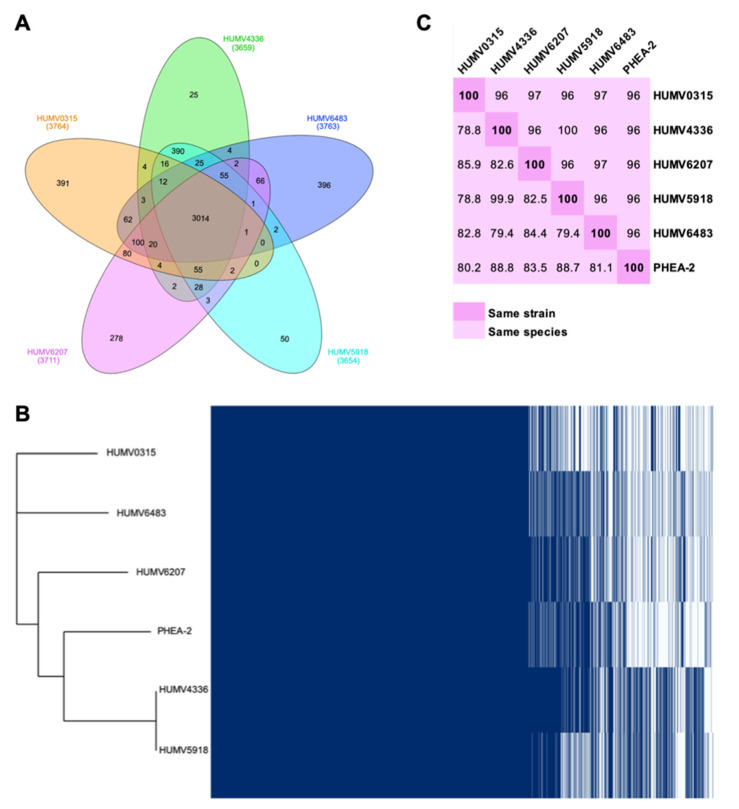
Pan-genome and taxonomic relationship of *A. pittii* isolates. (**A**) Venn diagram with the pan-genome composition of five *A. pittii* clinical isolates; in the middle, core-genome size. (**B**) Matrix of presence/absence genes generated with Roary software and the phylogenetic tree of 5 clinical isolates and a reference strain. (**C**) Matrix of evolutive distances in *A. pittii* strains. The upper part of the matrix shows ANI values; the lowest part of the matrix shows DDH values. ANI >95% and DDH >70% describe the same species; diagonal 100% shows the same strain.

Through the PanOCT tool, orthologous genes of the five clinical strains were determined, obtaining the pan-, core-, and accessory genomes of the group and each strain (Figure 1). In the Venn diagram from Figure 1A, the core-genome (3014 genes) and the accessory genome of each strain are shown, as well as the number of genes shared for each group of two to four isolates. Considering this information, it can be seen that the strain that shares a smaller number of genes with the group is HUMV6483, which has 396 unique genes, followed by HUMV0315 with 391. On the other hand, HUMV4336 and HUMV5918 are the most similar, due to sharing the higher number of CDS (390) between them and having the smallest number in their accessory unique genomes (25 and 50, respectively). With the information obtained from PanOCT, Roary software was also used to visualize the presence/absence of all the genes of the pan-genome as well as the phylogenetic tree using the reference strain PHEA-2 to confirm the species (Figure 1B). On that tree, the closer relationship between HUMV4336 and HUMV5918 was also observed, as well as HUMV0315 being taxonomically the farthest from the group.

For the taxonomic relationship among the isolates, besides the phylogenetic tree, both ANI (average nucleotide identity) and DDH (DNA–DNA hybridization) distances were obtained, also including the reference strain (Figure 1C). In the first analysis, 95% is considered the threshold to belong to the same species, while >70% is the threshold for DDH values. Strains HUMV5918 and HUMV4336 were the closest ones, with a value for ANI of 100% and DDH of 99.9%, due to the second one being more restrictive and, therefore, more accurate to differentiate among strains. Furthermore, the reference strain showed the highest DDH values for those two strains, as well as in Figure 1B, being phylogenetically closest to both isolates.

### 2.3. Genomic Annotation and Point Mutations

**Figure 2:**
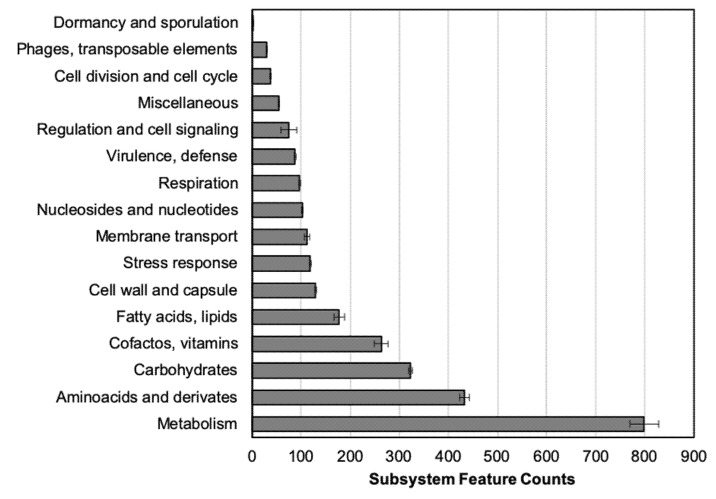
Number of protein-coding sequences distributed in each subsystem category according to RAST annotation. Each bar shows the average of 5 clinical strains ± SE (standard error).

RAST annotation classifies the predicted genes into subsystems (Figure 2). The highest amount of CDS belongs to “Metabolism” and molecular compounds (i.e., carbohydrates) subsystem groups. The group of antimicrobial-resistance determinants or related genes are included in the “Virulence, defense” subsystem, from 88.5 to 104 genes (average 95.8 ± 3.04), a number stable among all the tested isolates.

Along with RAST, Prokka annotation was also performed, and through the different databases, many determinants of resistance were identified (Table 3). The strain HUMV6483 was the only one that encoded an aminoglycoside-modifying enzyme, *ant(3*″*)-IIa*. All the strains also harbored one β-lactam class C gen (*ampC*) from four different allotypes, and at least two oxacillinases (*bla*_OXA-213_ was present in all the genomes, and HUMV0315 also harbored *bla*_OXA-58_), being the most common allotype *bla*_OXA-325_, present in the five isolates. Because HUMV0315 is the only meropenem resistant strain, *bla*_OXA-58_ might be responsible for that phenotype.

Besides the genetic determinants, antimicrobial resistance is sometimes related to point mutations in the antibiotic targets. For this reason, the main targets for fluoroquinolones and colistin were manually analyzed to map the point mutations. Among the five clinical strains sequenced, both *gyrA* (DNA gyrase) and *parC* (topoisomerase IV) amino acid sequences were compared for mutations in the quinolone-resistance-determining regions (QRDR). For both genes, HUMV0315 harbored a substitution of serine (S) by leucine (L) in positions 81 (DNA gyrase) and 84 (topoisomerase), *Acinetobacter* numbering, being the only strain that exhibited resistance against ciprofloxacin.

Regarding colistin resistance, mutations in the components of the operons *pmrAB* and *lpxACD* were analyzed. Due to all the tested strains being resistant to colistin, the reference *A. pittii* PHEA-2 was used as the sensitive control for mapping against [21]. Among the tested genes, two substitutions were found in the two-component regulator genes *pmrA* and *pmrB* in the clinical strains compared with the reference one. The five clinical strains showed a replacement 164Val→Ile in *pmrA* and 442Ile→Thr in *pmrB*. Among the other operon components, only *lpxA* showed a specific different sequence, between positions 201 and 210, where PHEA-2 harbored two substitutions in 201 (Met→Phe) and 202 (Arg→Lys) and deletion from 203–210. Other modifications were found among the analyzed sequence but with no correlation with the phenotypes, which points to the variability among the LPS structures (data not shown).

### 2.4. Plasmid Prediction and Characterization

In the four strains PacBio sequenced, two showed the presence of a plasmid structure, HUMV6207 of 100.37 kb and HUMV6483 of 112.60 kb, and only chromosomes without plasmid in the strains HUMV4336 and HUMV5918.

**Figure 3:**
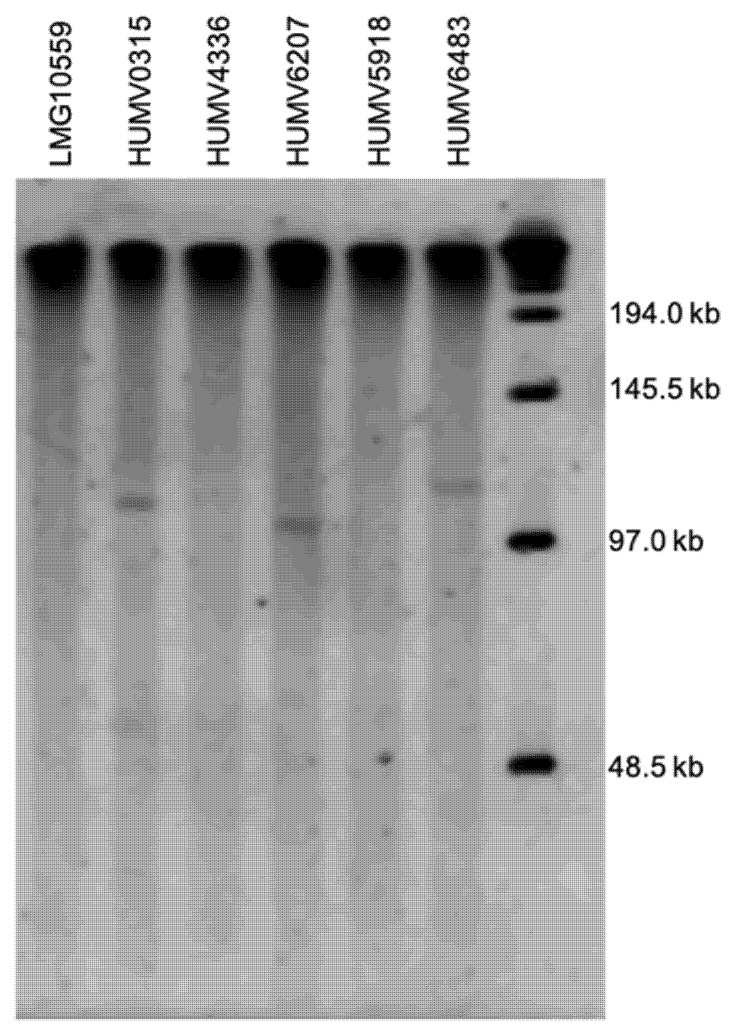
Molecular PFGE detection of plasmid in *A. pittii* strains. Molecular size ladder (Lambda ladder PFGE marker, NEB) on the right lane.

The PFGE analysis using S1 digestion in the five clinical *A. pittii* strains and the reference isolate LMG10559 is shown in Figure 3. Three out of the six strains were positive for a plasmid of a size between 97 and 145.5 kb, with the one in HUMV6207 being the smallest, and the one from HUMV6483 being the largest. On the other three strains, there were no bands that correspond with those structures.

With the specific primers designed by Bertini et al. [22], only the strain HUMV0315 showed a positive result, with a plasmid belonging to the homology group three (GR3) of *A. baumannii.*

**Figure 4:**
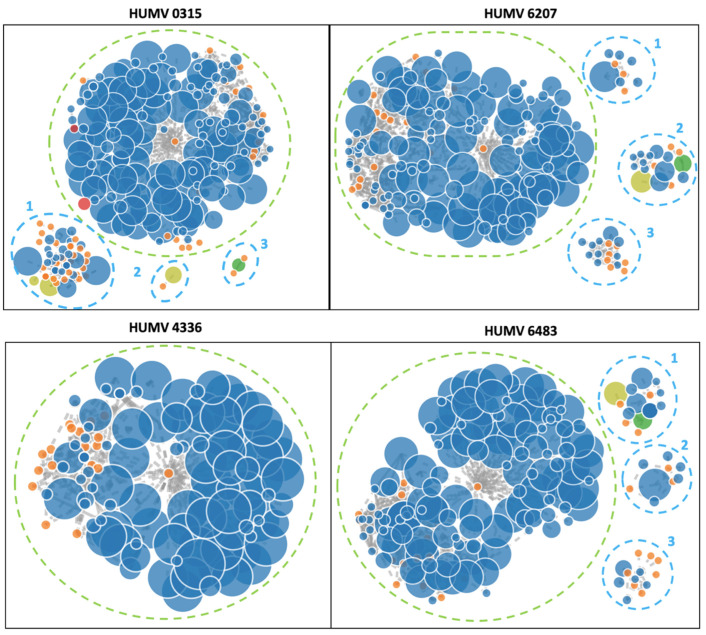
PLACNETw reconstruction of *A. pittii* draft genomes. Contigs are shown in blue nodes and in orange the reference genomes. The contigs node’s sizes are proportional to their length. The other color nodes represent contigs with specific proteins: yellow-green with RIP (replication initiation protein), red with REL (relaxases), and green with both. The dotted blue line shows the rounded plasmid predicted structures and the green-dotted lines show the chromosomes.

In the four genomes sequenced with Illumina, the software PLACNETw was used to identify the contigs that may belong to a plasmid (Figure 4). Only the strain HUMV4336 showed no presence of a plasmid, with a single chromosome of 3.9 Mb. The strain HUMV0315 had a chromosome of 3.9 Mb and three predicted plasmids of 115.88 kb (1), 9.33 kb (2) identified as pOIFC032-8.6 from *A. baumannii*, and 6.14 kb (3). The strain HUMV6207 also showed a single chromosome of 3.87 Mb and three putative plasmid structures of 35.72 kb (1), 59.78 kb (2), and 13.5 kb (3). Finally, the strain HUMV6483 showed a chromosome of 3.86 Mb and three groups of contigs predicted as plasmids of 58.97 kb (1) and 35.72 kb (2) with high identity with pMS32-1 and 12.45 kb (3).

### 2.5. PubMLST

Three out of the five clinical strains were classified in specific *A. baumannii* ST, as follows: HUMV0215 ST1023, HUMV6207 ST1818, HUMV6483 ST2083, as well as the reference strain PHEA-2 as ST1527. The other two strains were classified with the nearest profile, that was ST1914 for both of them, due to novel alleles for some of the genes. All the genes showed for these two strains 100% coverage, and 100% identity for genes *cpn60*, *gyrB*, and *rpoB*. The other genes showed different identity values, all over 98%: *gdhB* 98.5465%, *gltA* 99.5858%, *gpi* 98.3607%, and *recA* 99.4609%. These results highlight both the high genomic identity between *A. baumannii* and *A. pittii* and the lack of genomic information about the latter one.

## 3. Discussion

Interest in *Acinetobacter* spp. genomes is rising, while comparative analysis including other species than *A. baumannii* is scarce, involving the lack of information on the pathogenic *A. pittii* [2,23,24,25]. When we performed this analysis on our clinical strains through the panOCT software, we obtained a pan-genome of 4154 genes, distributed between the core-genome of 3014 genes, and accessory single genomes from 25 (HUMV4336) to 396 (HUMV6483) genes (Figure 1A). The large number of genes on the core-genome compared with the pan-genome highlights the high similarity among the sequenced strains. These data are in accordance with other studies performed in *Acinetobacter* spp., where the core-genome ranges from 2000 to 3000 genes even with a difference in sample sizes from 3 to 69 genomes [26,27,28,29,30].

Among the analyzed strain, HUMV4336 and HUMV5918 were found to be the closest between them, with ANI and DDH values of 100% and 99.9%, respectively, with both of them lacking any plasmid structure on their genomes. ANI distances are established for less restrictive values than DDH ones, which are always higher (Figure 1C) and could lead to a misidentification or consider two isolates as the same strain. These strains have been studied phenotypically and have shown that they have only slightly different properties in terms of virulence and survival onto solid surfaces [13,31].

In this work, the RAST tool was also used to classify the predicted CDS from the sequenced genomes into subsystems based on their function [32]. In all the strains, the main number of genes with described function belongs to “Metabolism” (protein, RNA, DNA, aromatic compounds, phosphorus, sulfur, and nitrogen) (Figure 2). This distribution is similar to the previous one observed in *A. baumannii*, while more genes are enclosed in this group in *A. pittii* [30]. Those results have been previously related to the fact of bacteria being pathogenic, where the number of metabolism-related genes is higher in bacteria with environmental niches than in the pathogenic ones [23,33]. In other work comparing different *Acinetobacter* species, authors highlighted their role as nosocomial pathogens, in which more similarities between *A. baumannii* and *A. pittii* were found than with other less clinically relevant species from the same complex, such as *A. nosocomialis* [2].

The *Acinetobacter* genus, mainly the species *A. baumannii*, has aroused much interest for the fast and apparently easy ability of its strains to acquire new resistance mechanisms. This phenomenon has also been seen, while it is less studied, in the other pathogen species *A. pittii*. According to that, in this work we have annotated and predicted different resistant determinants in clinically isolated strains of *A. pittii*, using for those purposes different databases to identify resistance genes, point mutations, and efflux pumps presence in their genomes. As shown in Table 3, only one strain harbored an AME encoding gene, but with a sensitive profile against gentamicin, which shows that this gene is not linked with the resistance to this specific aminoglycoside compound. In all the strains, a cephalosporinase encoding gene (*ampC*) was detected, with a broad diversity of allotypes among them. The most prevalent isoform, present in HUMV4336 and HUMV5918, highlighting again their genomic similarity, was ADC-25 which has been previously described [34,35]. A total of four allotypes were identified in the five strains, with all of them being resistant to a high concentration of ampicillin.

Regarding the oxacillinases, all the strains showed at least two different isoforms on their genomes. The five strains harbored the gen *bla*_OXA-325_, which encodes for an OXA-213 isoform, described for the first time in *A. calcoaceticus* [36]. Four out of the five strains, except HUMV0315, also harbored another isoform of OXA-213, *bla*_OXA-421_, which was more recently described for A. *pittii* [37]. In the HUMV0315 strain, two OXA-58 isoforms were identified, *bla*_OXA-58_ and *bla*_OXA-97_, considered as low-activity carbapenemases [38]. This strain was the only one resistant against meropenem, suggesting a link between that enzyme and the observed phenotype.

Regarding the efflux pump components encoding genes, in all the strains operons for the multiple efflux pumps from RND (resistance-nodulation-cell division) superfamilies *adeFGH* and *adeIJK* were found, which are able to target tetracycline and fluoroquinolones. AdeIJK has also been described as able to accommodate β-lactam and macrolide compounds [39,40]. Most of the strains also harbored the regulators *adeL* (but HUMV6207) and *adeN*, responsible for the overexpression of *adeFGH* and *adeIJK*, respectively [40]. The *adeABC* operon was only partially found in most of the strains (except for HUMV6207 that did not harbor any subunit), with the encoding genes for subunits AdeA and AdeB, and the regulator operon *adeRS* was missing in all the isolates. However, it is described that these two subunits are enough to be functional as long as they couple with another outer membrane channel. The efflux pump AdeABC (or AdeAB) is able to remove the majority of the antimicrobial compounds [39,40,41]. Another two efflux pumps, described for *A. baumannii*, were present in all the sequenced genomes: the SMR (small multidrug resistance) pump encoded by *abeS* whose substrate is macrolides; and the *abeM* that encodes for a MATE (multidrug and toxic compound extrusion) pump whose main target is fluoroquinolones [39,41].

Insertion sequences, as well as other mobile genetic structures such as transposons, are DNA segments that can change their location by themselves because they carry one or two transposase genes, whose main clinical relevance is that they are commonly linked to antimicrobial resistance genes and can transfer those elements between plasmid and chromosomes [42,43]. Different insertion sequences were found in all the isolates. In the strain HUMV0315, the insertion sequenced IS*Aba125* was found, described for the first time in *A. baumannii* with its role in the inactivation of an outer membrane porin [44]. More recently, the same sequence in *A. pittii* has been associated with the *bla*_OXA-58_ gen duplication and related to the presence of the *bla_NDM_*_-1_ gene [4,45]. This structure presence is usually related to the presence of some genes such as *bla_NDM_*, *bla*_OXA_, *ampC*, or *aph(6)*, hence playing a role in resistance against β-lactam or aminoglycosides [28,46,47]. In the strains HUMV4336 and HUMV5918, a high homology with IS*Aba53* from *A. baumannii* was found [48]. The strain HUMV6483 harbored the IS*Aba46* that was recently identified in *A. baumannii* [49]. Finally, the strain HUMV6207 harbored the element IS*Acsp1* from the Tn3 family described for *Acinetobacter* spp., whose presence has been more recently linked with conjugative plasmid in *A. baumannii* [50].

Moreover, other important genomic structures that could be also associated with mobile genetic elements and horizontal genic transfer are the genomic islands (GI) [51]. All the genomes were analyzed for those structures and all of them showed the presence of several predicted GI with sizes that range from 4 to 40 kb. Most of the annotated genes on them were associated with some specific metabolic process, transporters, transposases, and phage-like proteins. Further relevant annotated features were toxin–antitoxin systems (i.e., *higA* and *higB* in HUMV0315; *hipA* in HUMV6207), siderophores and ferric-metabolic systems (i.e., putative TomB system in HUMV5918 and HUMV0315), and some antibiotic modifying enzymes, such as *eryG* in HUMV0315, which encodes for an erythromycin 3′′-*O*-methyltransferase. All the predicted features are listed in Supplementary Material File S1.

Although the main resistance mechanisms in these bacteria are usually associated with hydrolytic enzymes or efflux pumps, other mechanisms such as the point mutations in antimicrobial targets also contribute to the MDR phenotypes. This is the main resistance mechanism against quinolones, whose clinical targets are DNA gyrase subunit A (*gyrA*) and topoisomerase IV (*parC*) [39,52]. In *Acinetobacter*, as in most bacteria, these mutations are located in the quinolone resistance-determining regions (QRDR) [30,39,53]. Among the strains studied here, online HUMV0315 showed substitution of 81Ser→Leu in the product of *gyrA*, as well as 84Ser→Leu in topoisomerase IV, linking these results with its resistance against ciprofloxacin. The mutations in *gyrA* and *parC* were described in *A. baumannii* for the first time in 1995 and 1997, respectively [54,55]; and more recently they have been described in *A. pittii* [53]. However, although these substitutions and the resistance phenotypes are well established, there is usually a mistake when comparing results among authors because of the use of *E. coli* as an alignment reference, which moves the mutation positions. The most recent works are already using each species numbering, as we have done in this work for *A. pittii*, pushing a consensus about this methodology.

While point mutations in *lpxACD* and *pmrAB* operons were analyzed for colistin resistance in *A. baumannii* [56], the authors did not find any recent publications with the same analysis of *A. pittii*. The most recent in silico analysis of *A. pittii* XDR colistin resistance only focused on the presence of the plasmid-encode *mcr-1* gene [25]. None of the clinical isolates used in this study harbored that gene, while many were colistin-resistant, which suggests that other mechanisms should be involved in that phenotype, as it happens to *A. baumannii*. Because all the used strains in this work were colistin resistant, the sensitive reference strain PHEA-2 was used to map the point mutations against both operon sequences. In the two-component regulator genes *pmrAB* involved in the lipid A synthesis [57,58], single substitutions in both genes were found, which are common in all the resistant strains: 164Val→Ile in *pmrA* and 442Ile→Thr in *pmrB* (*A. pittii* numbering). The *lpxACD* operon participates in the LPS biosynthesis, which is the cellular target of the polymyxin family [56]. There was only one replacement common for all the resistant strains in *lpxA* between positions 201 and 210, in which the sensitive strain harbored the substitution 201 (Met→Phe) and 202 (Arg→Lys), and deletion from 203–210. Due to there being no previous data on point mutations in these genes for *A. pittii*, this is the first report of specific substitutions that could be related to the resistant phenotype of *A. pittii* against the last-resort antibiotic colistin.

Although *A. baumannii* is a relevant pathogen in medical facilities, plasmid information availability about this species is scarce and mostly inexistent for *A. pittii* [30]. In this work, the identification and characterization of plasmids were conducted through PFGE and sequencing. Specific primers designed for an *A. baumannii* resistance plasmid were also used to analyze the similarity among these structures, due to *A. pittii* being the closest genomic species [22]. Using the PFGE profiles as previously described for *A. baumannii* [30], the presence of one (HUMV6207, HUMV6483) or two (HUMV0315) plasmids was found. There is no information about this technique used for plasmid in *A. pittii*, while it has been used for *Acinetobacter* chromosomes identification [59,60]. This approach has the limitation of the plasmid size detection because it is not sensitive enough for small plasmids, while it is well known that these bacteria can harbor plasmids from 2 kb to over 100 kb [61]. Through molecular identification with the specific *A. baumannii* primers, only one strain was positive, HUMV0315 in homology group three, which highlights the high homology between these species’ genomes both in chromosome and plasmid structures.

The PacBio SMRT (single-molecule real-time sequencing) was used with some of the strains, as this approach allows to obtain a close genome from the sequenced isolate, and hence predicts the presence of plasmids [62]. Two strains were positive for plasmid presence, HUMV6207 (100.37 kb) and HUMV6483 (112.60 kb). When comparing those results with the ones obtained from PFGE they correlate because these strains showed a single band over a 97 kb ladder band, with the second product being bigger than the former (Figure 3). The HUMV4336 and HUMV5918 showed only chromosomic DNA when sequenced, and were also negative in the PFGE assay.

Finally, isolates also sequenced through the Illumina platform were analyzed with the PLACNETw software (Figure 4). Although this software requires manual filtering and analysis and the sequencing results could be fragmented, making the prediction more difficult, accurate results were obtained. Strain HUMV4336 did not show any contig linked to a plasmid, in accordance with data from the PacBio and PFGE results. Strain HUMV0315 showed a plasmid of over 110 kb (upper band of PFGE) and other smaller structures that could be part of the same sequence. One of the structures showed homology with plasmid pOIFC032-8.6, recently described in *A. baumannii* [63]. Based on the PCR results, one of the structures may belong to the GR3, which could be identified as the *A. baumannii* plasmid but not included in the original classification because it was later described. A similar situation was found in strains HUMV6207 and HUMV6483, in which PLACNETw predicted three plasmids per genome, between 10 and 60 kb approximately, but with only one product of over 97 kb in the PFGE pattern. In those strains, through SMRT technology, only one plasmid larger than 100 kb was identified, suggesting that the three predicted structures by PLACNETw must belong to the same plasmid. One of the HUMV6483 showed high identity with pMS32-1 plasmid, recently described in *A. pittii* [64]. The use of different approaches would be so useful to study the genomic composition of the pathogenic bacteria, especially when the genomic information of species such as *A. pittii* is so scarce.

Both the genetic and phenotypic comparison of the two close species could be helpful, not only to describe and understand new features in *A. pittii*, but also to decipher and comprehend how *A. baumannii* became a most resistant and successful pathogen due to its high genomic identity.

## 4. Materials and Methods

### 4.1. Bacterial Strains

Five clinical *A. pittii* strains isolated from different patients (e.g., sputum, wound, and urine samples) at the Hospital Universitario Marqués de Valdecilla (HUMV) from Santander, whose phenotypic characteristics were already described [31], were used in this study. Reference strains LMG 10559 (also named RUH 509) [65] and PHEA-2 were also included [66].

### 4.2. Antimicrobial Susceptibility Assays

Antimicrobial susceptibility assays for seven compounds from the 6 main antibiotic families were performed by microdilution broth method, following CLSI guidelines [67]. Isolates were classified as resistant or susceptible according to the EUCAST breakpoints for *Acinetobacter* spp. or general no species-specific breakpoints, and CLSI breakpoints when no value was described in EUCAST [67,68]. Antimicrobials selected were tested at the following ranges: ampicillin 1–64 mg/L, meropenem 0.25–6 mg/L, colistin 0.125–8 mg/L, ciprofloxacin 0.06–4 mg/L, erythromycin 2–128 mg/L, gentamicin 0.5–32 mg/L, and tetracycline 0.5–32 mg/L.

### 4.3. DNA Isolation, Sequencing, and Assembly

Genomic DNA was isolated and purified from the five *A. pittii* clinical strains, using a GeneJET genomic DNA (gDNA) isolation kit (Thermo Scientific, Carlsbad, CA, USA), from overnight cultures incubated at 37 °C with shaking.

The gDNA was submitted to Fisabio (Valencia, Spain) for Illumina sequencing, where DNA libraries were generated through the Nextera XT Illumina protocol (catalog number FC-131-1024), and the multiplexing step was performed with the Nextera XT index kit (catalog number FC-131-1096). Finally, libraries were sequenced using a 2 × 300 bp paired-end run (MiSeq v3 reagent kit (catalog number MS-102-3003) on a MiSeq sequencer according to the manufacturer’s instructions (Illumina, Valencia, Spain)). Once sequenced, both paired and unpaired reads were used for the whole-genome sequence using Unicycler v0.3.0.b software [69]. 

The gDNA was also submitted to Macrogen (Seaoul, Korea) for PacBio single-molecule real-time (SMRT) sequencing, where a single library was prepared for each strain and run on one SMRT cell, providing almost 100% coverage of the genomes. After, the generated reads were introduced into the Hierarchical Genome Assembly Process version 3 (HGAP3), which includes assembly with the Celera Assembler and polishing with Quiver [70].

The quality of the sequenced products was analyzed using the FastQC server (version 0.11.9) with contig sizes in Illumina-sequenced genomes that ranged from 35 to 300 bp.

### 4.4. Genomic Annotation and Plasmid Prediction

The de novo assembled genomes were annotated using both rapid annotations using subsystems technology (RAST) server and software Prokka v.1.13 [71,72]. An MLST profile for the used strains was obtained through the server https://cge.cbs.dtu.dk/services/MLST/ (accessed on 31 March 2022) [73,74].

Antimicrobial determinants, as well as point mutations, were identified using different software to allow a higher and more accurate prediction as follows: ABRicate 0.8 software (https://www.github.com/tseemann/abricate/ (accessed on 31 March 2022) (Resfinder 3.2 and PointFinder databases)) [75], CARD database [76], and MEGA7 software for point mutation [77].

Plasmid in silico prediction was performed using the PLACNETw server [78]. The predictions results were correlated with characterization through PCR-based replicon typing (PBRT) with *A. baumannii* specific plasmid primers [22] and identification with pulse-field gel electrophoresis (PFGE) digestion approach as previously described [30]. Briefly, bacteria were treated overnight with lysozyme and incubated after with proteinase K for 16–20 h. They were digested with 20 U of S1 for 30 min and electrophoresis was performed in a CHEF-DR^®^ III system (Bio-Rad) for 22 h in total (6 h pulse from 1 to 15 s, and 16 h with 15 to 35 s).

Insertion sequences were predicted using the ISFinder database through the software https://www-is.biotoul.fr/blast.php (accessed on 31 March 2022) [79] and the platform IslandViewer (http://www.pathogenomics.sfu.ca/islandviewer/ (accessed on 20 April 2022)) was also employed to predict the genomic islands on the different genomes [51].

### 4.5. Pan-Genome Analysis

Identification of the species and evolutionary distances among the strains were calculated using ANI [80] and DDH [81] values, with PHEA-2 strain as reference and specie positive control [65]. Pan-genome analysis was performed through PanOCT [82] and Roary [83] software, as well as InteractiVenn for diagram creation [84].

## Figures and Tables

**Table 1:** List of MICs (mg/L) of each antimicrobial (top) for each *A. pittii* strain (left), and their resistance profile (right column). COL (C) colistin; MER (M) meropenem; AMP (A) ampicillin; GEN (G) gentamicin; CIP (P) ciprofloxacin; TET (T) tetracycline; and ERY (E) erythromycin. MIC, minimum inhibitory concentration; MIC_50_, MIC that inhibits 50% of the isolates; MIC_90_, MIC that inhibits 90% of the isolates. * No specific value for *Acinetobacter* spp.; general with no species-specific breakpoints used. ** CLSI breakpoints used.

		COL	MER	AMP *	GEN	CIP	TET **	ERY	Resistance Profile
MIC	LMG10559	8	2	>64	4	>0.25	1	>128	CAE
	HUMV0315	4	>16	>64	4	>4	1	64	CMAPE
	HUMV4336	8	2	>64	4	>0.125	1	>128	CAE
	HUMV6207	4	2	>64	16	>0.25	<0.5	64	CAGE
	HUMV5918	8	2	>64	1	<0.06	1	64	CAE
	HUMV6483	8	2	>64	2	<0.06	1	64	CAE
MIC_50_		>8	2	>64	2	0.125	1	64	
MIC_90_		>8	>16	>64	16	>4	1	>128	
Range (mg/L)		0.125–8	0.25–16	1–64	0.5–32	0.06–4	0.5–32	2–128	

**Table 2:** Features of *A. pittii* genomes according to RAST and Prokka annotation. * PacBio sequenced. CDS, coding sequences.

		HUMV0315	HUMV4336	HUMV6207	HUMV5918 *	HUMV6483
Size (bp)		4,040,319	3,936,103	3,970,495	3,990,911	4,008,138
% GC		38.8	38.8	38.8	38.9	38.9
Subsystems		448	454	459	308	452
CDS	RAST	3791	3664	3725	3827	3777
	Prokka	3777	3651	3697	3678	3770
tRNA		63	63	63	74	64
mRNA		1	1	1	1	1
rRNA		3	3	3	18	3

**Table 3:** List of antimicrobial resistance genes and antimicrobial efflux pump genes identified with Prokka and CARD databases in clinical *A. pittii* strains.

Strains	Antimicrobial Classes Resistance Genes			Efflux Pumps		
	AME	β-LACTAM		RND	SMR	MATE
		*ampC*	OXA			
HUMV0315		ADC-18	OXA-58 OXA-58 (97) OXA-213 (325)	*adeAB* *adeFGH* *adeIJK*	*abeS*	*abeM*
HUMV4336		ADC-25	OXA-213 (325) OXA-213 (421)	*adeAB* *adeFGH* *adeIJK*	*abeS*	*abeM*
HUMV6207		ADC-43	OXA-213 (325) OXA-213 (421)	*adeFGH* *adeIJK*	*abeS*	*abeM*
HUMV5918		ADC-25	OXA-213 (325) OXA-213 (421)	*adeAB* *adeFGH* *adeIJK*	*abeS*	*abeM*
HUMV6483	*ant(3″)-IIa*	ADC-19	OXA-213 (325) OXA-213 (421)	*adeAB* *adeFGH* *adeIJK*	*abeS*	*abeM*

## Data Availability

Not applicable.

## Supplementary Materials

The following supporting information can be downloaded at: https://www.mdpi.com/article/10.3390/antibiotics11050676/s1, File S1: List of all the predicted features.
